# Lack of Glutamate Receptor Subunit Expression Changes in Hippocampal Dentate Gyrus after Experimental Traumatic Brain Injury in a Rodent Model of Depression

**DOI:** 10.3390/ijms22158086

**Published:** 2021-07-28

**Authors:** Maxon V. Knott, Laura B. Ngwenya, Erika A. Correll, Judy Bohnert, Noah J. Ziemba, Emily Allgire, Tracy Hopkins, Jennifer L. McGuire

**Affiliations:** 1 University Honors Program, University of Cincinnati, Cincinnati, OH 45221, USA; knottmv@mail.uc.edu; 2 Department of Neurosurgery, University of Cincinnati College of Medicine, Cincinnati, OH 45267, USA; erikacorrell@umass.edu (E.A.C.); bohnerjy@ucmail.uc.edu (J.B.); NOZ6@pitt.edu (N.J.Z.); morttl@ucmail.uc.edu (T.H.); kozioljl@ucmail.uc.edu (J.L.M.); 3 Department of Neurology and Rehabilitation Medicine, University of Cincinnati College of Medicine, Cincinnati, OH 45267, USA; 4 Neuroscience Graduate Program, University of Cincinnati, Cincinnati, OH 45267, USA; johns4ey@mail.uc.edu

**Keywords:** depression, hippocampus, traumatic brain injury, glutamate receptor, Wistar Kyoto

## Abstract

Traumatic brain injury (TBI) affects over 69 million people annually worldwide, and those with pre-existing depression have worse recovery. The molecular mechanisms that may contribute to poor recovery after TBI with co-morbid depression have not been established. TBI and depression have many commonalities including volume changes, myelin disruption, changes in proliferation, and changes in glutamatergic signaling. We used a well-established animal model of depression, the Wistar Kyoto (WKY) rat, to elucidate changes after TBI that may influence the recovery trajectory. We compared the histological and molecular outcomes in the hippocampal dentate gyrus after experimental TBI using the lateral fluid percussion injury (LFPI) in the WKY and the parent Wistar (WIS) strain. We showed that WKY had exaggerated myelin loss after LFPI and baseline deficits in proliferation. In addition, we showed that while after LFPI WIS rats exhibited glutamate receptor subunit changes, namely increased GluN2B, the WKY rats failed to show such injury-related changes. These differential responses to LFPI helped to elucidate the molecular characteristics that influence poor recovery after TBI in those with pre-existing depression and may lead to targets for future therapeutic interventions.

## 1. Introduction

Traumatic brain injury (TBI) affects over 69 million people annually worldwide and is a major cause of disability [1,2]. Recovery after TBI can be affected by many pre-existing conditions, such as psychiatric disorders. Individuals suffering from depression are at increased risk for TBI and experience prolonged recovery after injury [3,4,5]. It has been shown that patients who report moderate to severe symptoms of depression prior to injury report greater severity of behavioral and cognitive problems post-injury, as well as a lower mental-health related quality-of-life [6]. Little is known about the underlying molecular mechanisms that predispose patients with pre-existing depression to poor outcomes after TBI.

Major depressive disorder (MDD) is associated with significant histological and biochemical changes, especially in the hippocampus. Specifically, these changes are associated with hippocampal dysfunction, which may interfere with recovery and lead to worse outcomes [7]. Patients with MDD have been shown to have smaller hippocampal volumes [8,9,10], decreased new cell generation [11,12], and abnormal neurotransmitter balances [13]. In addition to the well-known monoamine theory of depression, which has been the basis of the selective serotonin reuptake inhibitor antidepressant medications, glutamate signaling has become a recent target for anti-depressive treatments. Studies have found lower levels of glutamatergic neurons in the hippocampus of MDD patients [14]. Recently, it has been demonstrated that glutamate N-methyl-D-aspartate (NMDA) receptor blockades, such as by ketamine, can be used for acute depressive crises [15,16,17].

TBI produces a similar glutamatergic signaling environment as MDD [18]. Glutamate can be present in large amounts in acute phases after TBI, with significant deficits in the chronic phase [19,20,21]. In addition, electrical pathology known to contribute to worse outcomes after TBI, namely spreading depolarizations, are propagated by the diffusion of glutamate [22,23,24]. Spreading depolarizations are episodes of extensive neuronal depolarization resulting in loss of neuronal ion homeostasis and a period of electrical silencing [25]. This activity spreads across the contiguous cortex and can be induced in many brain regions, including the hippocampus [26]. These spreading depolarizations have been shown to be a prominent pathology in patients with TBI and prognosticate a worse outcome [27,28,29]. Spreading depolarizations are blocked by NMDA receptor antagonists [30,31,32,33] because of their dependence on glutamate. A glutamate balance is critical in the hippocampus, as it plays an important role in learning and plasticity through NMDA receptors and exhibits excitotoxic properties in excess levels [34]. Thus, changes in the glutamatergic environment after TBI may contribute to poor cognitive outcomes.

There is a lack of knowledge as to how pre-existing depression may contribute to changes in glutamatergic signaling after TBI. To investigate whether there are subacute glutamate receptor changes after TBI that are unique to pre-existing depression, we used a well-established experimental model of TBI, the lateral fluid percussion injury model (LFPI), and an animal model of depression.

The Wistar Kyoto (WKY) rat has been demonstrated behaviorally and molecularly to be an ideal model for depression. Phenotypically, they have shown increased susceptibility to stress ulcers, less active open field activity, and less mobile forced swim tests [35]. The WKY strain also exhibits molecular signatures of depression, such as decreased evoked glutamate release, lower initial concentration of GluN2A and GluN2B subunits, lower NMDA receptor binding, and reductions in the hippocampal and cortical volumes [36]. The WKY strain also exhibits responses to antidepressant medications, with a reversal of behavioral and molecular phenotypes with some medications [36,37,38].

We investigated glutamatergic signaling in the rat model of depression (WKY) compared with its parent Wistar strain (WIS) after a moderate LFPI. We examined the glutamate receptor subunit expression in the hippocampal dentate gyrus, as we have previously shown changes in glutamate signaling in the hippocampus and because of the now recognized importance of this neurotransmitter system in both TBI and depression [20,21,26]. In addition, we also examined hippocampal volume and cell proliferation, as these are known histopathological features of MDD. Rather than explore the acute changes that occur immediately after injury, which are inherently intertwined with the inflammatory response, we chose to evaluate a subacute time point three weeks after injury. Our long-term goal is to establish what changes occur as a result of injury in this subacute timepoint, such that interventions can be derived that address this post-injury milieu. We hypothesized that TBI would differentially impact glutamate signaling in the WKY strain after TBI. Here, we show that WIS rats showed changes in the glutamate receptor subunit expression after TBI, yet WKY failed to show such changes, which may represent an inappropriate injury response and a target for intervention in TBI with co-morbid depression.

## 2. Results

### 2.1. WKY Animals Show Deficits in Behavior Consistent with a Depression Phenotype

**Figure 1:**
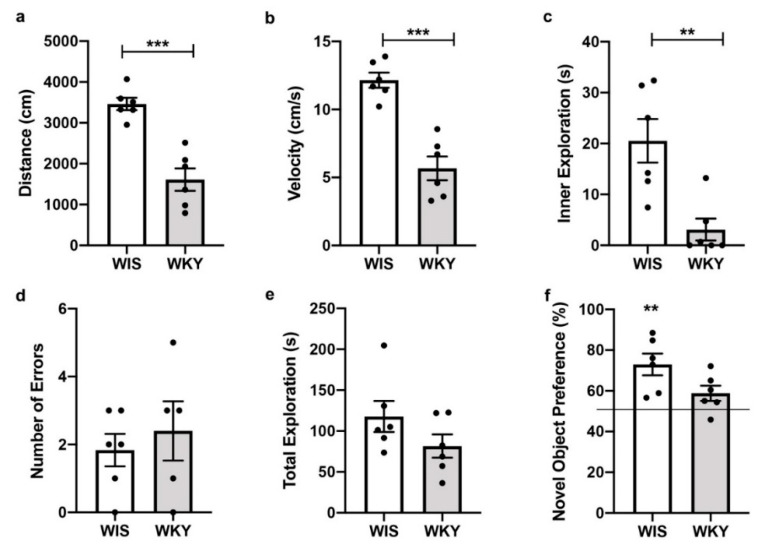
WKY animals show a behavioral phenotype consistent with depression. In the open field arena, WKY animals (**a**) travelled a shorter distance, (**b**) had a slower velocity, and (**c**) spent less time exploring the inner portion of the arena. In the working memory task, radial arm maze, (**d**) and WIS and WKY had an equivalent number of errors. In the short-term memory task, for novel object recognition, although WIS and WKY (**e**) spent a similar amount of time exploring objects, (**f**) WIS showed a statistically significant preference for the novel object, whereas WKY did not show a preference for the novel object. ** *p* < 0.01, *** *p* < 0.001.

To demonstrate a depressed phenotype in the WKY strain, the WIS and WKY animals not subject to injury procedures were tested behaviorally. Open field results showed that naïve WKY animals had a significantly lower total distance traveled (t(10) = 5.894, *p* < 0.001; Figure 1a) and slower velocity (t(10) = 6.254, *p* < 0.001; Figure 1b). WKY animals also spent less time exploring the inner portion of the arena (Mann−Whitney U *p* = 0.0087; Figure 1c), consistent with an anxiety/depression phenotype.

In the working memory task, a radial arm maze, animals were required to visit all 8 arms of a maze within 10 min. The maze consisted of eight concentric arms that radiated from a central base. Revisiting a previously visited arm was recorded as an error. The WKY animals were not statistically different in the number of errors from WIS animals (t(9) = 0.5981; *p* = 0.5645; Figure 1d). However, in the short-term memory task, novel object recognition (NOR), WKY had similar total exploration times (t(10) = 1.528, *p* = 0.158; Figure 1e), yet did not perform better than chance on novel object preference (one-sample *t*-test versus chance (50%): WIS t(5) = 4.305, *p* = 0.0077; WKY t(5) = 2.358, *p* = 0.0649; Figure 1f).

### 2.2. Similar Injury Phenotypes in WIS and WKY

**Figure 2:**
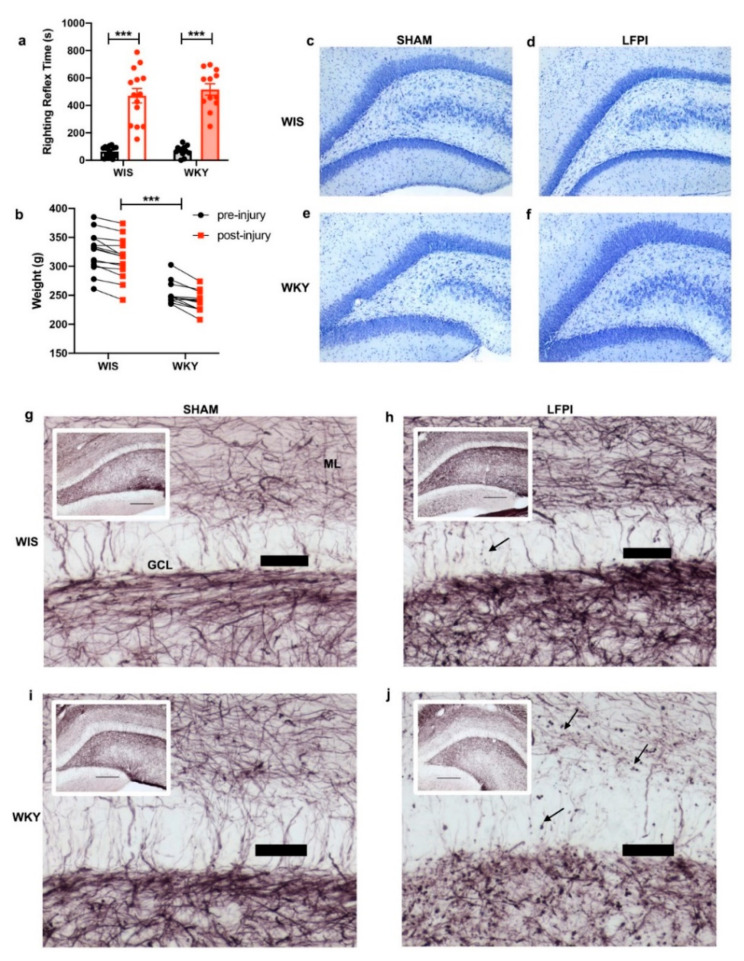
Equivalent injuries were produced in WIS and WKY animals, although WKY showed exaggerated myelin changes. (**a**) Injured animals (red bars) in both the WIS and WKY group had increased righting reflex times immediately after injury compared with the sham (black bars) animals. There was no statistically significant effect of strain. (**b**) All of the injured animals showed a decrease in weight on the post-injury day. Repeated measured ANOVA showed a significant effect of injury and additionally an effect of strain. As illustrated, the age-matched WKY animals had significantly lower pre-injury weights. (**c**,**d**) The WIS animals did not show large lesions in the dentate gyrus with injury, nor did the WKY (**e**,**f**) animals. (**g**) The WIS sham animals showed a normal pattern of myelination in the dentate gyrus granule cell layer (GCL) and molecular layer (ML). (**h**) Three weeks post-injury the WIS animals showed signs of myelin loss, including areas of punctate staining (arrow). (**i**) The WKY animals also showed a normal myelination pattern. However, the WKY injured animals (**j**) showed an extensive decrease in myelin staining and exaggerated punctate staining (arrows). Insets (**g**–**j**) show low power (10× objective) images of gold chloride stained dentate gyrus, scale bar = 250 µm. Scale bars (**g**–**j**) = 50 µm. *** *p* < 0.001.

A moderate LFPI (mean atm 2.09 ± 0.17) was induced, with equivalent injuries being produced in the WIS and WKY animals. There was no interaction effect of injury plus strain in the reflex righting time (RRT); however, in both strains, sham animals had a low RRT whereas the injured group had a longer RRT with a statistically significant effect for the injury group (F(1,48) = 148.2, *p* < 0.0001; Figure 2a).

As expected with injury, both strains showed weight loss on post-injury day one. Using repeated measured ANOVA on the injured group, there was no effect of strain and injury (F(1,24) = 0.2336, *p* = 0.633). However, both groups showed statistically significant weight loss at day one post injury (F(1,24) = 56.03, *p* < 0.001; Figure 2b). This also illustrates that although age-matched, the WKY animals had lower starting weights, as there was an effect of strain (F(1,24) = 42.34, *p* < 0.001) that was also present in the sham animals (F(1,24) = 37.38; *p* < 0.001; Supplementary Material Figure S1).

Qualitatively, both the WIS and WKY animals showed minimal gross histological changes in the dentate gyrus, as demonstrated with Nissl staining using Cresyl Violet (Figure 2c–f), consistent with histological findings of a moderate LFPI. However, with gold chloride staining, which illustrates myelin changes, both strains showed qualitative differences consistent with injury. The WKY strain showed exaggerated punctate staining and loss of myelination in the hippocampal dentate gyrus with injury (Figure 2g–j).

### 2.3. AMPA Receptor Subunits Were Not Significantly Altered with Regards to Injury or Strain

**Figure 3:**
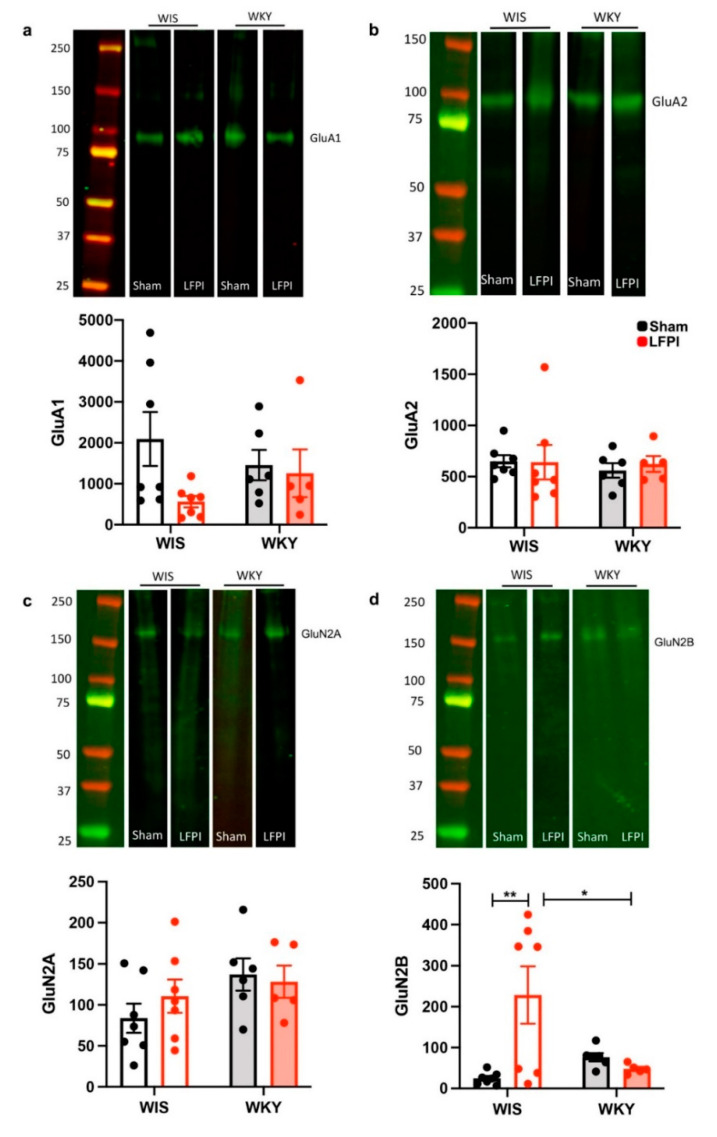
Changes in glutamate receptor subunit expression after injury, normalized to the total protein stain. (**a**) A representative selection of immunoblotting shows GluA1 protein expression with expected bands at 100 kD in all conditions. Normalized GluA1 protein expression in WIS animals (open bars) showed a decrease in GluA1 expression after injury (red bars), but did not reach statistical significance with two-way ANOVA. WKY animals (filled bars) did not show any changes in GluA1 protein expression with injury. (**b**) Representative immunoblotting shows GluA2 with expected bands at 100 kD in all of the groups. There were no differences appreciated in strain or injury in the normalized GluA2 protein expression. (**c**) Representative immunoblotting shows GluN2A with expected bands at 170 kD and no differences in the normalized protein expression between strain or injury condition. (**d**) A representative selection of immunoblotting is shown for GluN2B illustrating expected bands at 170 kD. Two-way ANOVA demonstrated a significant increase in the GluN2B normalized protein expression after injury in WIS but not in WKY. * *p* < 0.05, ** *p* < 0.01. Complete immunoblotting results are available in Supplementary Figure S2.

To determine the influence of injury on the AMPA receptor subunits, we performed immunoblotting on isolated dentate gyrus tissue. The AMPA glutamate receptor subunits, GluA1 and GluA2, did not show significant changes as a result of injury or strain at three weeks after injury. GluA1 showed no significance in either strain (two-way ANOVA, F(1,21) = 0.0036, *p* = 0.9526; Figure 3a) nor post-injury (ANOVA, F(1,21) = 3.220, *p* = 0.0872; Figure 3a), although the WIS animals showed a trend towards a decreased GluA1 expression with injury that did not reach significance in the two-way ANOVA analysis. The GluA2 expression showed no significance in either strain (ANOVA, F(1,24) = 0.3388, *p* = 0.5660; Figure 3b) nor effect of injury (ANOVA, F(1,24) = 0.1192, *p* = 0.7330; Figure 3b).

### 2.4. Divergent Changes in NMDA Receptor Subunit Expression with Injury

The effect of injury and strain on the NMDA receptor subunits was determined via immunoblotting on the isolated dentate gyrus tissue. GluN2A did not show any strain differences or changes with injury (interaction F(1,21) = 0.816, *p* = 0.376; Figure 3c). However, GluN2B showed an overall significant effect of the interaction of strain and injury (F(1,21) = 8.223, *p* = 0.0092; Figure 3d). Specifically, the WIS animals showed an increase in GluN2B expression with injury (WIS Tukey post-hoc sham versus LFPI *p* = 0.0053), while the WKY did not show a change with injury.

### 2.5. No Changes in the Dentate Gyrus Volume after Injury

**Figure 4:**
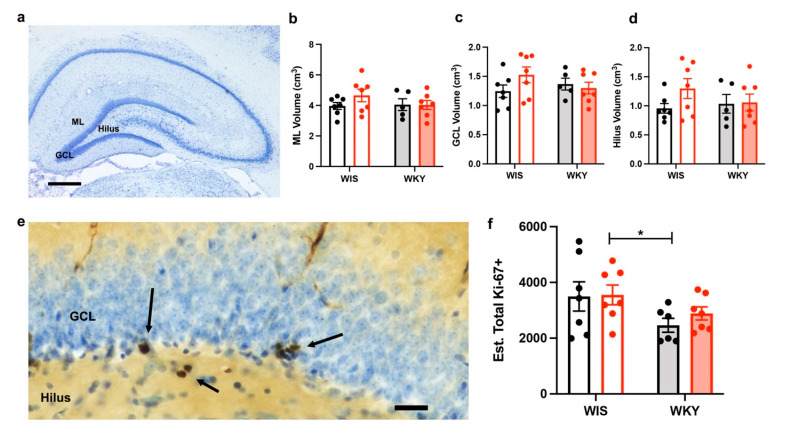
No dentate gyrus volume changes but fewer proliferative cells in WKY strain. (**a**) Cresyl violet stained sections were analyzed for volume of the molecular layer (ML), granule cell layer (GCL), and Hilus. No statistically significant findings were seen in the ML (**b**), GCL (**c**), or Hilus (**d**) between WIS (open bars), WKY (filled bars), or between the sham (black) or injured (red). (**e**) Ki-67 immunopositivity is illustrated in this example photomicrograph from a WIS sham animal. Ki-67+ nuclei are noted by brown diaminobenzidine (arrows). Sections were counterstained with Cresyl Violet. (**f**) Although there was no difference in Ki-67 immunopositivity three weeks after LFPI, there was an effect of strain, with WKY having fewer Ki-67+ cells. Scale bar in (**a**) = 500 µm, in (**e**) = 25 µm; * *p* < 0.05.

To assess whether strain and injury related volume differences in the dentate gyrus contributed to pathology, we performed an unbiased stereological assessment of the volume via the Cavalieri method. No dentate gyrus specific volume changes were detected between the strains or three weeks after injury. There was no difference in volume in the molecular layer (ML), granule cell layer (GCL), or Hilus of the dentate gyrus between WIS and WKY strain (F(1,22) = 0.64, 0.208, and 0.31, respectively, *p* > 0.1). There was also no volume loss with injury (F(1,22) = ML 0.995, GCL 0.835, and Hilus 1.59, *p* > 0.1; Figure 4a–d). The total hippocampal volume was also assessed and there were no strain or injury differences (Supplementary Figure S3).

### 2.6. WKY Animals Have Fewer Ki-67+ Cells but No Change with Injury

Because cell proliferation differences are a feature of both depression and TBI, we estimated the total number of proliferating cells using an unbiased stereological counting technique on tissue immunohistochemically stained for the proliferative cell marker Ki-67. Overall, there was no statistically significant effect of injury times’ strain interaction on the Ki-67+ cell counts in the dentate gyrus GCL and Hilus (F(1,23) = 0.2472, *p* = 0.6238). However, there was a strain effect (F(1,23) = 5.264, *p* = 0.031; Figure 4e,f), with the WKY animals having overall fewer Ki-67+ cells.

## 3. Discussion

In summary, we have shown that WKY animals showed deficits in cognition, exhibited a lack of GluN2B receptor subunit protein expression changes after injury, and demonstrated a low Ki-67+ cell count in the dentate gyrus when compared with the WIS strain, despite having no difference in the dentate gyrus volume.

The WKY has historically been a good model for depression, as the strain shows depressive-like responses to novel stress in emotional behavior testing [39]. Our results are consistent with previous studies showing WKY rats as being less active in the open field tests, spending less time in the center of the open field, and performing poorly in short term memory tasks [35]. Although the open field test has several drawbacks [40], it has consistently been shown to provide a measure of anxiety-like behavior and has been linked to depressed behavior [35,41,42,43]. Memory and cognitive deficits are also characteristic of depression. For example, spatial field memory deficits are seen in clinical tests involving patients with major depressive disorder [44]. Impaired spatial learning and impaired recognition memory have been shown in WKY rats [36]. We did not find a deficit in the radial arm maze, a working memory task. Others have shown conflicting results of working memory deficits in the WKY strain [45,46]. We did demonstrate a deficit in the short-term recognition task NOR. This is consistent with what others have shown as WKY strain related deficits [47].

Our behavioral testing paradigm was limited, and did not test classical tasks of depression such as the forced swim, tail suspension, and sucrose preference test. Although we did not independently assess these tasks in this study, others have demonstrated deficits in these tasks of depression in WKY [35,36], as well as data showing limited reversal of depression phenotypes with antidepressant medications [48,49].

We utilized this depressed phenotype WKY strain as a model of pre-existing depression in TBI, and are the first to show differentiated responses three weeks after LFPI. Both the WIS and WKY strains showed equivalent responses to LFPI, with no significant strain differences in weight loss or reflex righting time. However, we found qualitative differences in gold chloride staining. Gold chloride staining has been used to determine an anatomical analysis of nerve endings and myelinated axons [50,51]. Both the WIS and WKY animals showed signs of myelin loss in the dentate gyrus three weeks after LFPI. However, the WKY strain showed exaggerated myelination abnormalities post-injury, including loss of myelination in the molecular layer and punctate staining throughout. It has been shown that myelin abnormalities in the brain exist in patients with MDD [52]. Thus, our findings of qualitatively decreased gold chloride staining in the dentate gyrus is are with WKY as a reliable model of depression. Additionally, our data support that of others, showing white matter changes as a consistent pathology after TBI [53,54,55]. We found indicators of decreased myelination after LFPI in both WIS and WKY rats. Our data suggest that with pre-existing depression, as in the WKY rats, exacerbated changes in the myelin composition may contribute to the injury pathophenotype. At the subacute three-week after injury timepoint that we examined, it is unknown if these qualitative myelin changes represent myelin in a state of potential recovery or irreversible damage. Further quantitative analysis of myelin changes after TBI, including evaluation for microglial activation and oligodendroglial myelin biogenesis, may help to elucidate the effect of pre-existing depression on myelin related pathology after injury and to identify the relevant targets for intervention.

In addition to qualitative differences in myelination between strains, we further demonstrated differential expression patterns in the glutamatergic signaling system. We are the first to show a lack of glutamate receptor subunit protein expression changes after LFPI in WKY animals. TBI can be characterized initially as causing a “storm” of glutamatergic signaling, with signaling dysfunction resonating from acute to chronic phases [20]. The environments in MDD and TBI are similar, with notable changes being decreased excitatory amino acid transporter expression, decreased AMPA expression, and a change in NMDA receptor subunit composition [18].

AMPA and NMDA receptors play key roles in memory and learning, and show differences in MDD and changes after TBI. After TBI, it has been shown that post-injury, the AMPA receptor subunit GluA1 decreases, and the GluA2 receptor subunit undergoes endocytosis [56]. Like TBI, depression in chronic stress models selectively decreases AMPA receptor expression with decreased GluA1 expression in the hippocampus of Sprague Dawley rats [57,58]. Our findings report a trend in decreased GluA1 expression in the WIS strain after LFPI that was not present in WKY. The lack of statistical significance with two-way ANOVA is likely as a result of insufficient power. Our previous immunoblotting studies suggested that six to eight animals per group would be sufficient to allow us to detect differences. However, as experimental TBI has not previously been performed in the WKY strain, data to influence our study design were limited. Despite an underwhelming amount of literature on the investigation of AMPA receptor changes with response to stress in preclinical models, differential results have been found. Atkins and colleagues published a study demonstrating the upregulation of GluA1 post-TBI in the hippocampus of Sprague Dawley rats at the acute timepoint [59]. While it has been reported that the GluA1 expression decreases at chronic timepoints in the hippocampus after a model of severe TBI [60], at our subacute timepoint of three weeks post-injury, we suggested a trend of decreasing GluA1 in WIS but not WKY. Our findings did not show any changes or trends in GluA2 expression in either strain or injury. This aligns with a previous study finding no changes from chronic stress on GluA2 expression in the hippocampus of Sprague Dawley rats [58]. Yet, although not in the hippocampus, in-vitro and in-vivo studies have found that TBI increases GluA2 internalization and relocalization [61,62]. The antibody used in the current study addressed the total GluR2 expression. It is known that AMPA receptors contain the edited GluR2(R) subunit, unedited GluR2(Q) subunit, or lack the GluR2 subunit; these changes drastically affect calcium permeability. Unlike NMDA receptors, most AMPA receptors in the brain are calcium impermeable, yet calcium permeable AMPA receptors are found in synapses that are susceptible to synaptic plasticity and disease [63]. The role of unedited and edited GluR2 subunits in learning and memory has not been addressed in TBI with the WKY model and should be investigated in future studies, as calcium permeability is important in learning and memory. Overall, the AMPA receptor subunit expression after experimental TBI has not been thoroughly characterized, and further investigations are warranted.

In contrast, there is abundant literature on the NMDA receptor subunit expression in both TBI and MDD. NMDA receptors have long been shown to be critical in memory formation and play an important role in neurogenerative diseases [64]. This purported role of injury related glutamate excitotoxicity has also made NMDA receptors popular targets for study. We did not find differences in the GluN2A subunit expression in strain or injury. However, we did find a significant increase in GluN2B expression in WIS rats three weeks after LFPI. The GluN2A:GluN2B ratio is of great interest, as injury and stress drive the subunit composition to favor the GluN2B subunit. It has been reported that GluN2A predominant receptors are synaptic, while GluN2B predominant receptors are extra synaptic [65]. Additional differences in the receptor subunits include that GluN2B is especially vulnerable to mechanical stress, such as TBI. When upregulated, GluN2B can hinder the neuronal network remodeling response to plasticity [66]. Increases in GluN2B have also been shown to be related to neurodegenerative pathways in diseases such as Parkinson’s, Alzheimer’s, and Huntington’s disease [67]. Whether the increase in GluN2B after experimental TBI is related to the later development of post-TBI neurodegeneration is unknown. Our results show that the WIS experienced a significant increase in GluN2B expression, but the WKY failed to elicit a change in expression. The lack of response in the WKY rat suggests that the ability of the glutamatergic signaling system to react to additional stress of TBI is deficient. Lei and Tejani-Butt found decreased NMDA receptor bindings via autoradiographic methods in the hippocampus in response to chronic stress in both WIS and WKY, with the WKY having a significant difference in response compared with the WIS [68].

Overall, our data demonstrate that WKY showed a lack in ability to respond through glutamatergic signaling in response to injury, however there are some important limitations to our findings. The demonstrated difference in WIS GluN2B expression after TBI was detected by immunoblotting. Immunoblotting has traditionally been wrought with concerns regarding accuracy. Some of this concern about the accuracy of semi-quantitative immunoblotting is in regards to the practice of normalizing proteins of interest to housekeeping proteins. In injury, it is unclear if the level of housekeeping proteins truly remains stable, therefore we used a validated approach of normalizing to the total protein stain to help ensure accuracy in our results [69,70]. However, even with this improved technique for quantitative immunoblotting, our results were limited in spatial resolution. We collected dentate gyrus tissue for the immunoblotting analysis. Although this narrowed the cytoarchitectural region of interest, our results must be interpreted with an understanding of the lack of cell level specificity. An increase in GluN2B expression could represent an increase in expression on post-synaptic neuronal membranes, however increased production of the GluN2B subunit without expression on the surface membranes is possible, as is an increased expression on the astroglial membranes or other extrasynaptic sites. We utilized only whole homogenates and not synaptosomes or other advanced molecular localization techniques, thus our findings suggest avenues for future research. In addition, we chose to examine a subacute time-point of three weeks after injury, because of our interest in non-inflammatory changes in the glutamatergic system after TBI. We found significance only in the GluN2B subunit at this time point, however the trajectory of changes over time was not explored. It is possible that acutely after injury there are a variety of glutamatergic signaling changes as part of the initial inflammatory response after injury. As there is a deficit in literature investigating the relationship between using a preclinical model of depression and investigating the changes in the glutamatergic signaling system in response to TBI, further investigation is required to better understand the signaling environment. Post-injury behavioral data were not obtained as we did not want a potential stress response associated with behavior testing to interfere with our molecular outcomes. However, future studies incorporating post-injury behavior testing may allow for a better understanding of the implications of these receptor subunit changes.

In addition to the changes in the neurotransmitter environments, depression has another pathology with similarity to TBI, such as tissue volume and cell proliferation changes. It has been reported in both preclinical and clinical situations that hippocampal volume is decreased in TBI and MDD. Proliferation and changes in neurogenesis also occur in response to stress and TBI.

A decrease in hippocampal volume has been demonstrated in patients with MDD, however, our study found no difference in dentate gyrus volume in the depressed WKY strain. We also did not find a difference in volume with injury. A previous study used the Cavalieri method and successfully found decreases in the hippocampus volume in the WKY in comparison to the Sprague Dawley strain, but did not report findings specifically to the dentate gyrus [71]. A smaller hippocampal volume in the WKY strain has also been demonstrated using magnetic resonance imaging [72], however the animals were weight-matched not age-matched, which may have contributed to the differences observed. Our study did not find a difference in volume when comparing the WKY to age-matched WIS controls using an unbiased stereological assessment, and additionally suggests that injury-related volume loss is not present three weeks after a moderate LFPI. Of note however, we did not count the individual neuron number in the dentate gyrus or hippocampal subfields, nor did we specifically assess for dying neurons. Our results do not preclude that there may be injury related cell specific changes, or ongoing cell death that is present, but not appreciated, in our assessment of regional volume.

We are the first to look at the Ki-67 cell counts in the dentate gyrus in the WKY compared with the parent strain of the WIS. One prior study investigated Ki-67 immunopositivity in the WKY hippocampus, comparing it to the spontaneous hypertensive rats (SHR) and stroke prone SHR (SHRSP) rats [73]. This study found significantly less Ki-67 positive cells in the WKY strain compared with the SHR rats. We found decreased cell proliferation in the WKY strain compared with the WIS strain, but no effect of injury. The decrease in proliferation by strain was expected based on other studies that have shown that the WKY exhibit attenuated neurogenesis measured by BrdU and doublecortin immunopositivity compared with the WIS in the dentate gyrus [74]. The lack of change with injury was not unexpected, as our prior data at two weeks after LFPI in the Sprague Dawley strain showed that the acute increases in cell proliferation after injury [75,76] had in fact decreased at two weeks after injury [77]. Others have also shown a return to baseline proliferative capacity two to three weeks after injury [78,79]. Our data are consistent with the literature, which suggests that the initial increase in the proliferative capacity after experimental TBI is followed by a return to baseline, and possibly a depletion of active stem cells may contribute to this decrease [80,81]. As Ki-67 counts reflect cell proliferation at the single timepoint of animal sacrifice, limitations exist when making conclusions about the survival and phenotype of newly generated cells. There are likely differences related to the animal strain, mechanism of experimental TBI, and injury severity that dictate the time course of changes in proliferative activity after injury.

In conclusion, our study showed a lack of GluN2B increases in the dentate gyrus in the WKY strain after experimental TBI. Our study provides supporting evidence that anatomical, morphological, and neurochemical changes in the dentate gyrus may contribute to a differential response to injury in pre-existing depression. Further research dictating the extent of this differential response may help contribute to targeted treatments for TBI patients with pre-existing depression.

## 4. Materials and Methods

### 4.1. Experimental Animals

We used 64 age-matched Wistar (WIS) and Wistar Kyoto (WKY; Charles River) rats, randomly assigned to naïve (*n* = 6/strain), sham (*n* = 12–14/strain), or LFPI (*n* = 12–14/strain). The average weight for the WIS rats was 322.1 ± SD 35.1, and for WKY was 252.8 ± SD 18.0. All of the animals were weighed pre-injury and daily after injury for the first week to assure maintaining an appropriate weight. All of the animals were in standard housing with a 12:12 h ratio of light to dark, and given ad libitum access to food and water, except for those that received the food restriction protocols described. All of the procedures were reviewed and approved by the Institutional Animal Care and Use Committee (IACUC).

### 4.2. Behavioral Testing

Naïve animals underwent behavioral testing to characterize their depressed phenotype. Because of the interaction between stress and depression, we did not behaviorally test animals that were assigned to undergo surgery and LFPI in order to avoid a potential confounder of our histological and molecular outcomes. Six animals per strain underwent behavioral testing. Figure S4 illustrates the experimental design for the animals undergoing behavioral testing. All of the animals were habituated to the testing environment and the tester with a habituation protocol that involved handling for ~5 min per day, travel to the behavioral testing suite, and the administration of food treats for one week prior to testing. The animals were food restricted during the behavioral testing battery. For food restriction, they were fed 5 g of food each day after completion of the behavioral testing, with daily weight measurement taken to assure animals did not lose ≥15% of their body weight.

#### 4.2.1. Open Field Activity Task

To assess the locomotor, depressive, and anxiety behavior, naïve WIS and WKY rats were allowed to explore a 50 gallon open arena (63 cm diameter). Distance travelled (in cm), velocity (cm/s), and time spent exploring the center of the arena (in seconds) were video recorded over a 5-min period using Ethovision Software and were confirmed by manual review of the video.

Time spent in the center of the arena, defined as the inner half (32 cm diameter), was calculated and scored as an indicator of anxiety/depression.

#### 4.2.2. Radial Arm Maze

Working memory was tested with the radial arm maze, as previously described [82,83]. In this task, the animals were habituated to a maze with eight concentric, evenly spaced arms radiating out from a central base. During habituation, all eight arms were food-baited and the animals were allowed free exploration for 15 min, repeated daily over a two-day period. On the day of the working memory task (day 3), the animals were placed in the center and given 10 min to visit all eight un-baited arms looking for a food treat. The optimal strategy involved visiting each arm only once. Visiting a previously entered arm was defined as an error; the number of errors prior to entering all eight arms was recorded. Data are presented as the total number of errors.

#### 4.2.3. Novel Object Recognition

To test the short-term memory, the rats were tested on a novel object recognition task within the open field arena. The animals were allowed to explore the arena for 10 min without any objects so as to re-familiarize them with the arena. The following day, the animals were placed in the arena for ten minutes with two identical objects affixed to opposite sides of the arena. After a 90 min interval, each rat was presented with one familiar object and one novel object, and was allowed to explore for 5 min. Time exploring objects was calculated by manual review of the recorded videos. Exploration time was counted only when the animal was facing the object and interacting with the object (grooming near the object or with their backside towards the object were not counted as exploration). Between novel and familiar trials and between each animal, all of the objects and the arena were cleaned with 70% ethanol to minimize olfactory cues. The total time spent exploring both objects was reported to assure that differences in short-term memory were not confounded by overall less exploration. The novel object recognition percentage was defined as the amount of time spent exploring the novel object ÷ total exploration time × 100.

### 4.3. Lateral Fluid Percussion Injury

Anesthesia was induced via 4% isoflurane and was maintained with 2–3% isoflurane throughout the surgical procedures. All of the procedures followed sterile techniques, including shaving the surgical site and sterilization with 70% alcohol and betadine solution. Craniectomy surgery and lateral fluid percussion injury were performed as previously described [77,83,84]. Briefly, the rats were mounted in a stereotaxic frame and a 4 mm diameter craniectomy was drilled with a trephine 4 mm posterior to the bregma and a 2.5 mm lateral of the sagittal suture, centered over the right parietal cortex. A plastic luer-loc hub was affixed over the craniectomy site with cyanoacrylate glue. A screw was inserted into the skull over the left parietal lobe, which provided support for the dental acrylic cement that was applied to maintain the hub until injury. The incision was sutured shut and the plastic hub was covered with a screw-on cap. The animals were given one dose of 0.03 mg/kg sustained release Buprenorphine (0.7–1 mg/kg, ZooPharm) subcutaneously for the post-op analgesia. The animals recovered from the procedure and lateral fluid percussion injury proceeded on day 3 after surgery. All of the animals received 5 min of anesthesia via the inhalation of 4% isoflurane, the cap covering the craniectomy hub was removed and the hub was filled with saline to remove air bubbles and create a watertight seal to the output valve of the LFPI device. The animals in the injured group received a fluid pulse to the dura at a pressure of 2 atm (2.09 ± 0.17). Sham animals were subjected to the same anesthetic and surgery protocols, but a fluid pulse was not delivered. After sham or injury, the animals were laid on their backs to assess time to spontaneously right (righting reflex time, RRT) as an index of injury severity. The animals were then briefly re-anaesthetized via the inhalation of 4% isoflurane, the surgical cement and hub were removed, and the surgical site was sutured closed with an absorbable suture. The animals with evidence of inappropriate injury at the time of tissue acquisition were not included in the study (one sham, two LFPI; additional detail in Supplementary Materials).

### 4.4. Tissue Acquisition

For immunohistochemical processing, the rats were administered 1 mL/kg of anesthetic (ketamine 100 mg/mL, xylazine 10 mg/mL) prior to perfusion fixation. The animals were randomly assigned to either fixation or fresh tissue harvest (Figure S4). The animals were transcardially perfused with a phosphate buffer, followed by 4% paraformaldehyde, and the brains were post-fixed in 4% paraformaldehyde overnight at 4 °C and then submerged in a graded sucrose solutions for cryoprotection. The brains were then flash frozen in cold isopentane and stored at −80 °C until use. The brains were sectioned in the coronal plane using a freezing-stage microtome at 40 μM thickness through the entirety of the hippocampus. The tissue was cut in six series and collected free-floating into vials of cryoprotectant and stored at −20 °C until they were batch processed for immunohistochemistry.

For immunoblotting, the rats were sacrificed via rapid decapitation using a guillotine. The brains were quickly removed and dissected in a cold phosphate buffer. The brains were hemisectioned along the sagittal suture, the thalamus and brainstem were removed, and the ipsilateral dentate gyrus was rapidly isolated with the aid of a 27 G needle tip and a dissecting microscope [77,85]. The tissue was immediately snap frozen on dry ice and stored at −80 °C until homogenized for protein analysis.

### 4.5. Immunohistochemistry and Histological Analysis

#### 4.5.1. Nissl Staining and Analysis of Dentate Gyrus Volume

The tissue sections representing one of every sixth section throughout the hippocampus were mounted on slides and processed for Cresyl Violet. The slides were washed, and then submerged in 0.1% Cresyl Echt Violet (ScyTek Lab #CEA999). The slides were then rinsed and subjected to subsequent dehydration in graded alcohol solutions, cleared in xylene, and coverslipped with permount.

Cresyl Violet stained sections from sham and injured WIS and WKY animals were analyzed for volume differences. A Zeiss Axio Imager M2 (Carl Zeiss Microscopy GmbH; Jena, Germany), equipped with a motorized stage, and controlled by StereoInvestigator software (MicroBrightField; Williston, VT, USA) was used to quantify the volume via the Cavalieri estimator probe. The dentate gyrus granule cell layer (GCL), hilus, and molecular layer (ML) were outlined at 10× magnification in every 24th section throughout the entire rostral–caudal extent of the dentate gyrus. Volumetric markings were conducted at a grid size of 50 µm. The estimated volumes were obtained for the dentate gyrus by an operator blind to the animal strain and injury group.

#### 4.5.2. Gold Chloride Staining

Randomly selected animals (*n* = 2 per injury group per strain) were stained with gold chloride to further characterize the injury. In a protocol modified from Schumed [86], sections were mounted on gelatinized slides and allowed to dry overnight before being submerged in 0.2% gold chloride (Fisher Scientific, Florence, KY, USA) in PBS for two hours, rinsed in distilled water, and treated for 5 min in 2.5% sodium thiosulfate (Fisher). After 30 min in several changes of tap water, the slides were dried overnight before clearing with xylenes and coverslipping with DPX. Sections containing dorsal hippocampus, centered at the craniectomy site (approximate stereotactic level of Bregma −3.0) were compared between the groups and strains for qualitative differences in myelin staining.

#### 4.5.3. Ki-67 Immunohistochemistry and Analysis

Ki-67 staining was conducted on a series of every 24th section spaced at 960 μM throughout the hippocampus of the sham as well as injured WIS and WKY animals. Antigen retrieval was performed using Tri-sodium citrate (pH 6) kept at 60 °C for 30 min. The sections were then blocked with 3% normal horse serum (NHS; H1270 Sigma-Aldrich, Inc., St. Louis, MO, USA) and 0.1% triton-x for 30 min at room temperature. The tissue was incubated overnight at 4 °C in the primary antibody solution consisting of mouse anti-Ki-67 (NCL-L-Ki67-MM1 Leica Biosystems, Buffalo Grove, IL, USA; 1:250), 3% NHS, and 0.1% triton-x. Secondary antibody incubation was performed for 1 h at room temperature with biotinylated horse anti-mouse (BA-2000 Vector Labs, Burlingame, CA, USA; 1:250), 3% NHS, and 0.1% triton-x. The tissue was quenched with cold 10% H_2_O_2_ in methanol prior to incubation in an avidin–biotin complex (PK-6100 Vector Labs, Burlingame, CA, USA). Sections were developed using a diaminobenzidine kit (SK-4100 Vector Labs, Burlingame, CA, USA) and were then mounted onto glass slides, counterstained with Cresyl Violet, and coverslipped.

The estimated total number of Ki-67+ cells was obtained for the dentate gyrus. The regions of interest included the granule cell layer and hilus, which were counted by an operator blind to the subject group and strain. The Ki-67 labeled nuclei were identified using a 100× oil objective on a Zeiss Axio Imager M2 (Carl Zeiss Microscopy GmbH; Jena, Germany), equipped with a motorized stage, and controlled by StereoInvestigator software (MicroBrightField; Williston, VT, USA). As Ki-67 labeling is rare, an optical fractionator with an exhaustive counting scheme including guard planes was used, and estimates of the total Ki-67+ cells were calculated using N = Q × 1/asf × 1/ssf × 1/tsf, as previously described [87].

### 4.6. Immunoblotting

The tissue was homogenized in an mPER and protease inhibitor cocktail (100×). Western blots were performed following a previously optimized protocol [26]. A BCA assay was conducted to determine the protein concentration of each sample. This determined protein concentration allowed us to standardize our samples, such that the appropriate amount of lysate was loaded to yield a final protein concentration of 15 μg of protein/well, which was loaded in 4–15% Mini-PROTEAN TGX precast gels (#4561086 Bio-Rad, Hercules, CA, USA). The samples were prepared with a loading buffer (6×) then denatured at 70 °C for 10 min before being loaded. The samples were run in a Tris-Glycine running buffer at 200 V for 30 min. The gels were then transferred onto membranes via wet transfer with a Tris-Glycine transfer buffer. A total protein stain was done prior to the primary antibody incubation in order to determine the normalization factor, as previously described [69,70,88]. The membranes were then incubated overnight at 4 °C in various primary antibodies. For the GluA1 and GluA2 mouse models, anti GluA1 and GluA2 monoclonal antibodies were used at 1:500 (Antibodies, Inc., Davis, CA, USA; RRID:AB_2315840 and AB_2232661, respectively). Mouse anti GluN2A and GluN2B antibodies at 1:250 were used (Antibodies, Inc., Davis, CA, USA, RRID:AB_2315842 and AB_10673405, respectively). The membranes were washed then incubated at room temperature in a red or green secondary polyclonal antibody used at 1:20,000 (LI-COR Biosciences Cat# 925-68072 and 926-32212, RRID:AB_2814912 and AB_621847, respectively). Finally, the membranes were imaged with an Odyssey Imager. The protein band signals were quantified using Image Studio (LI-COR) and the expression was normalized to the total protein stain.

### 4.7. Statistical Analysis

Statistical analysis was performed using GraphPad Prism 8.4.3 for Mac (GraphPad software). Behavioral results of the naïve animals were analyzed with an unpaired *t*-test after first checking for normality using a Shapiro–Wilk test. Data that did not pass the test for normality were analyzed with Mann–Whitney U. Novel object recognition data were additionally analyzed via a one-sample *t*-test with a theoretical mean (chance) of 50%. Data comparing WIS and WKY sham and LFPI animals (immunoblotting, cell counting, and volume data) were analyzed with a two-way ANOVA with Tukey’s post-hoc multiple comparisons test. Changes in weight at one day post-injury were evaluated using a repeated measures ANOVA. In all of the analyses, significance was identified by *p* < 0.05.

## Data Availability

The data presented in this study are available within the article and the contained Supplementary Materials.

## Supplementary Materials

The following supplementary materials are available online at https://www.mdpi.com/article/10.3390/ijms22158086/s1.
