# Supplementary material for: Lack of Glutamate Receptor Subunit Expression Changes in Hippocampal Dentate Gyrus after Experimental Traumatic Brain Injury in a Rodent Model of Depression
Source: Int J Mol Sci. 2021 Jul 28;22(15):8086. doi: 10.3390/ijms22158086 (PMC8347641; doi:10.3390/ijms22158086)
Supplement: Supplementary file 1 [file ijms-22-08086-s001.zip › ijms-1287245-supplementary.pdf]

Supplementary Material

Figure S1. Age-matched sham WKY animals have lower weights. There was no overall effect of strain x injury interaction in repeated measures ANOVA on the pre-injury (black) versus one day post-sham injury (red) weights [$F(1,24) = 0.3550$, $p=0.557$]. Both WIS and WKY strains did have a slight weight change on post procedure day 1 [$F(1,24)=7.316$, $p=0.012$]. Additionally, there was a strong significant effect of strain [$F(1,24)=37.38$, $p <0.0001$.

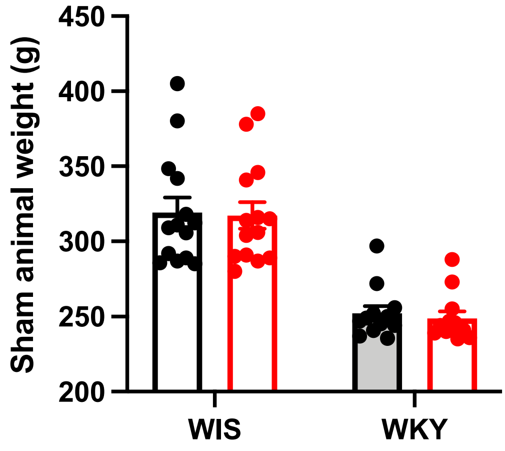

Figure S2. Complete immunoblotting results. Samples were run in duplicate and randomized across blots. All blots were run in the same sample order. The blot for GluN2a was re-probed without stripping for GluA2 to preserve sample. All membranes were probed for total protein (red) prior to immunoblotting and band intensity for each sample was normalized to its own loaded protein. The duplicates were then averaged for the final data point. In the panels below, W= WIS, K=WKY; S = Sham; T=TBI (LFPI).

(a) GluA1

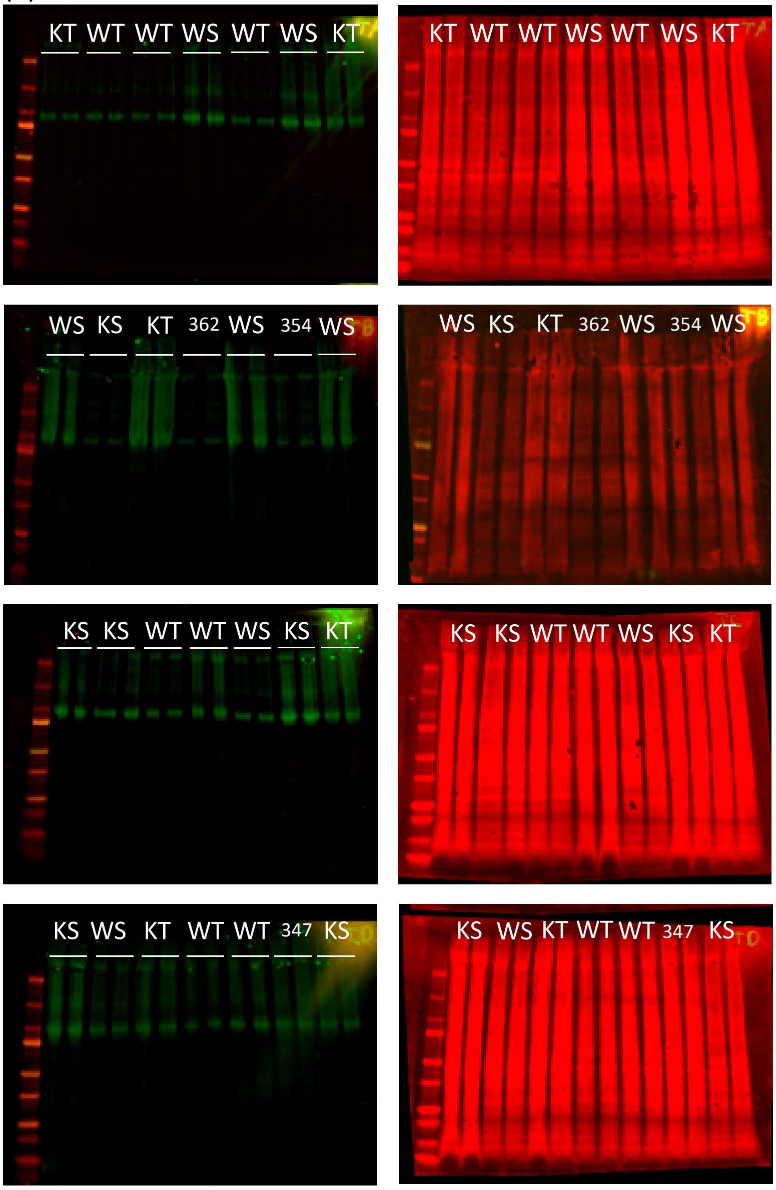

(b) GluA2

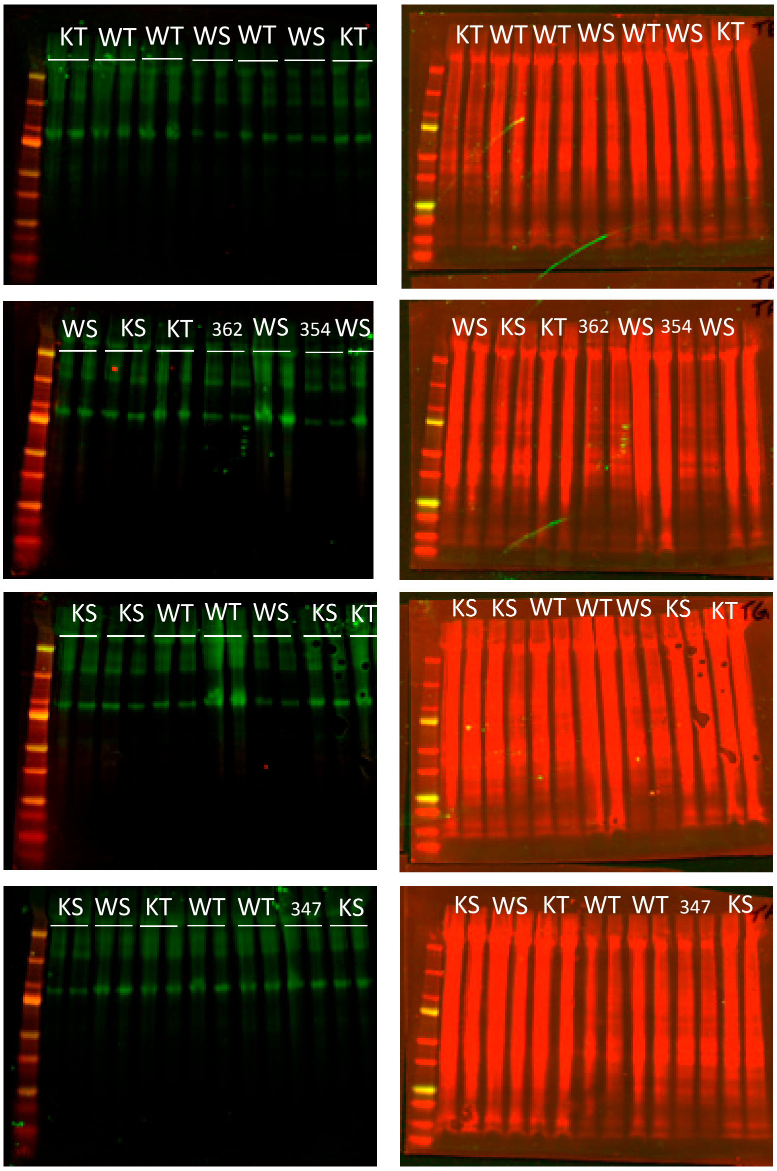
KT WT WT WS WT WS KT
KT WT WT WS WT WS KT
WS KS KT 362 WS 354 WS
WS KS KT 362 WS 354 WS
KS KS WT WT WS KS KT
KS KS WT WT WS KS KT
KS WS KT WT WT 347 KS
KS WS KT WT WT 347 KS

(c) GluN2a

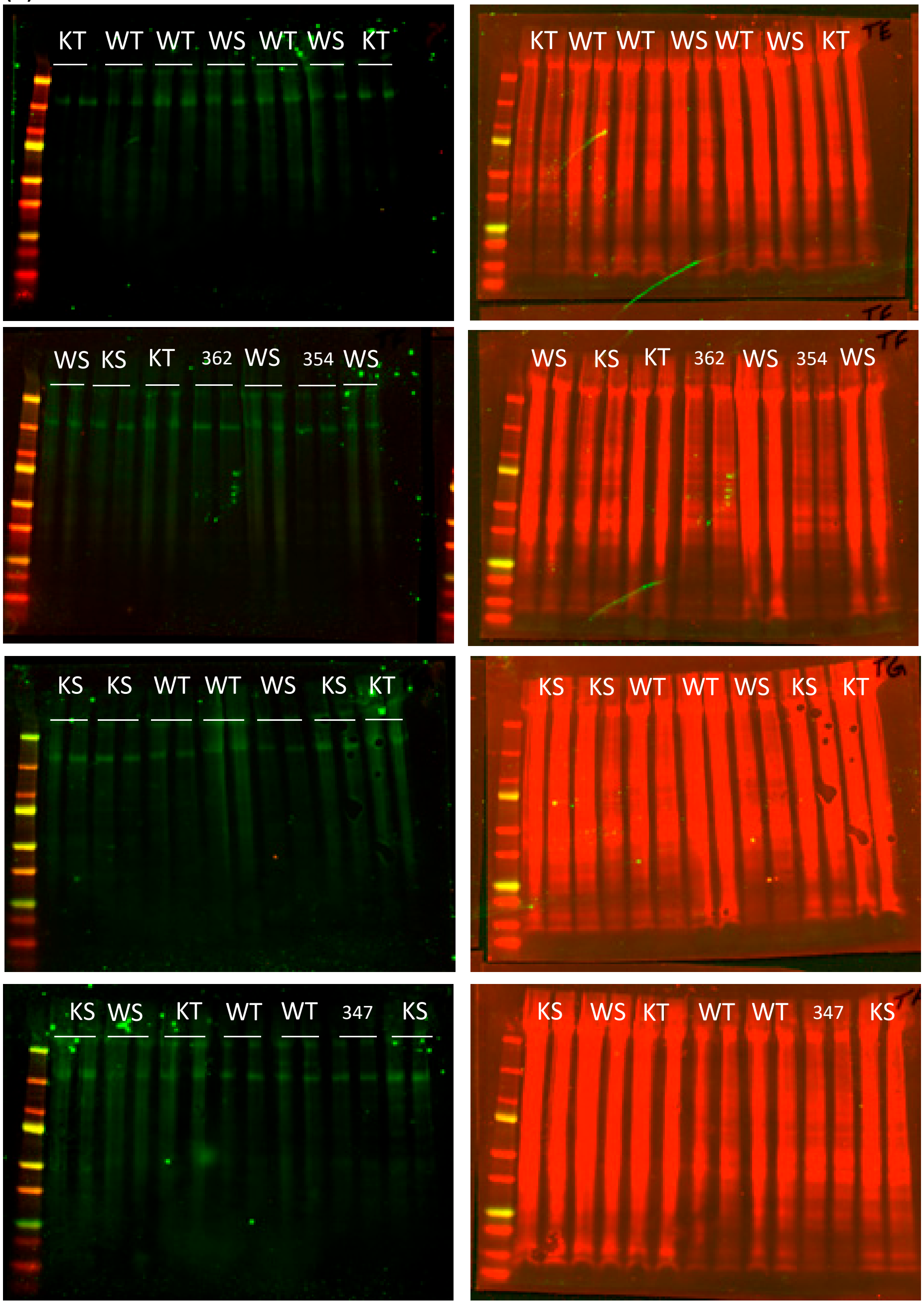

KT WT WT WS WT WS KT
KT WT WT WS WT WS KT
WS KS KT 362 WS 354 WS
WS KS KT 362 WS 354 WS
KS KS WT WT WS KS KT
KS KS WT WT WS KS KT
KS WS KT WT WT 347 KS
KS WS KT WT WT 347 KS

(d) GluN2b

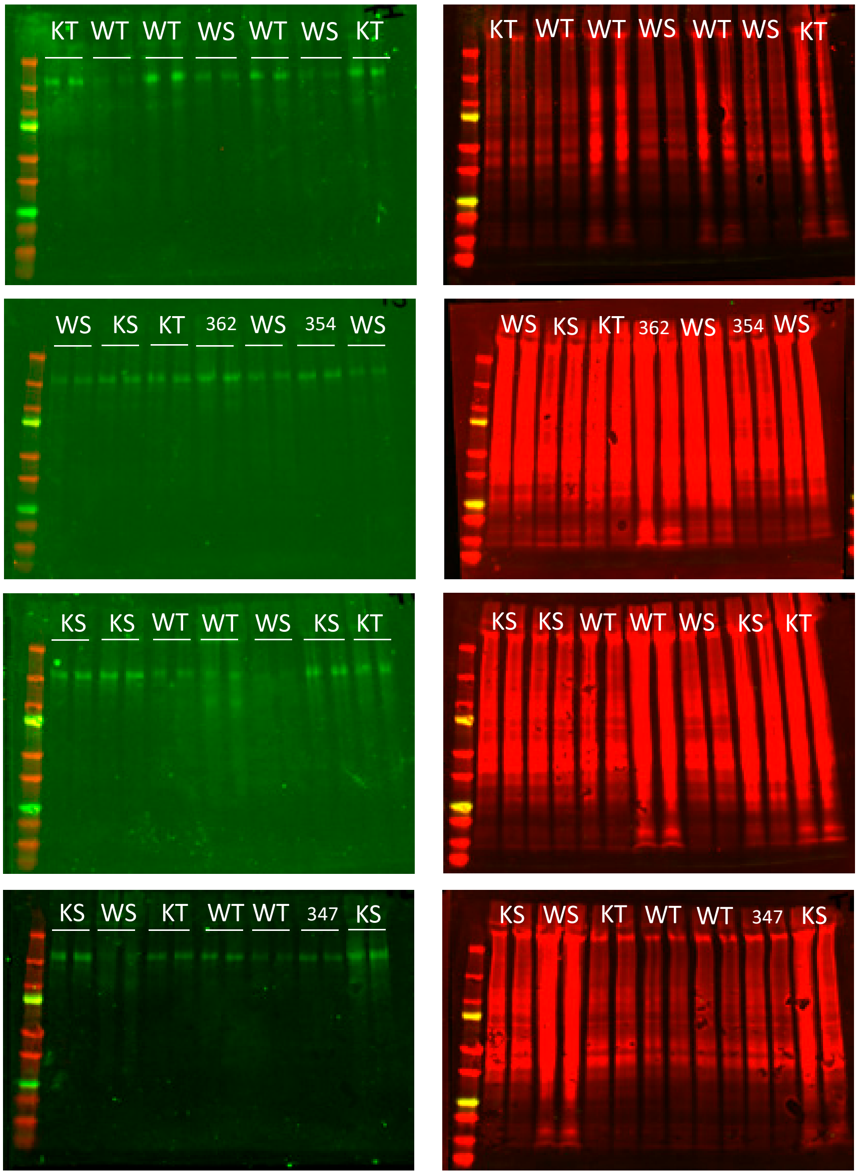
KT WT WT WS WT WS KT
KT WT WT WS WT WS KT
WS KS KT 362 WS 354 WS
WS KS KT 362 WS 354 WS
KS KS WT WT WS KS KT
KS KS WT WT WS KS KT
KS WS KT WT WT 347 KS
KS WS KT WT WT 347 KS

Figure S3: No change in total hippocampal volume with strain or injury. Total hippocampal volume was assessed using Caviliere method in age-matched WIS (open bars) and WKY (filled bars) sham (black) and injured (red) animals. There was no strain x injury effect by 2 way ANOVA [$F(1,22) = 1.2$, $p=0.272$]. There was also no independent effect of strain [$F(1,22)=0.6$, $p=0.420$] or injury [$F(1,22)=0.7$, $p=0.400$].

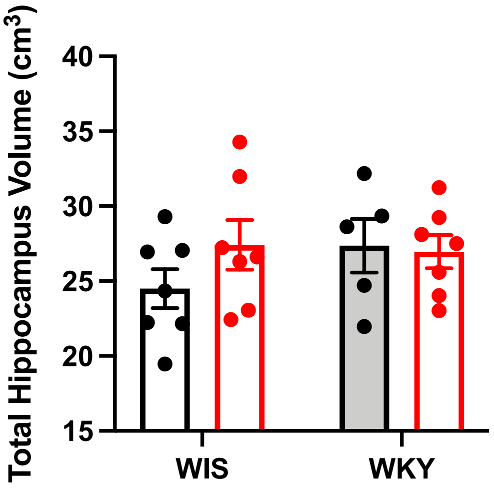

Figure S4: Timeline illustrating experimental design. (a) Naïve animals, not subject to anesthesia and surgical procedures were used to validate behavioral findings of a depression phenotype. Animals underwent testing for open field, novel object recognition, and radial arm maze after extensive habituation to both the environment and the behavioral tests. Behavioral testing was completed at a time point that correlated to 3 weeks after study end. (b) Animals that underwent surgical procedures for either sham or LFPI had n=12-14/strain per injury group. 3 weeks after injury, tissue was harvested and animals were randomly selected for fresh tissue dissection (for immunoblotting) or 4% paraformaldehyde fixation (for immunohistochemistry and histology).

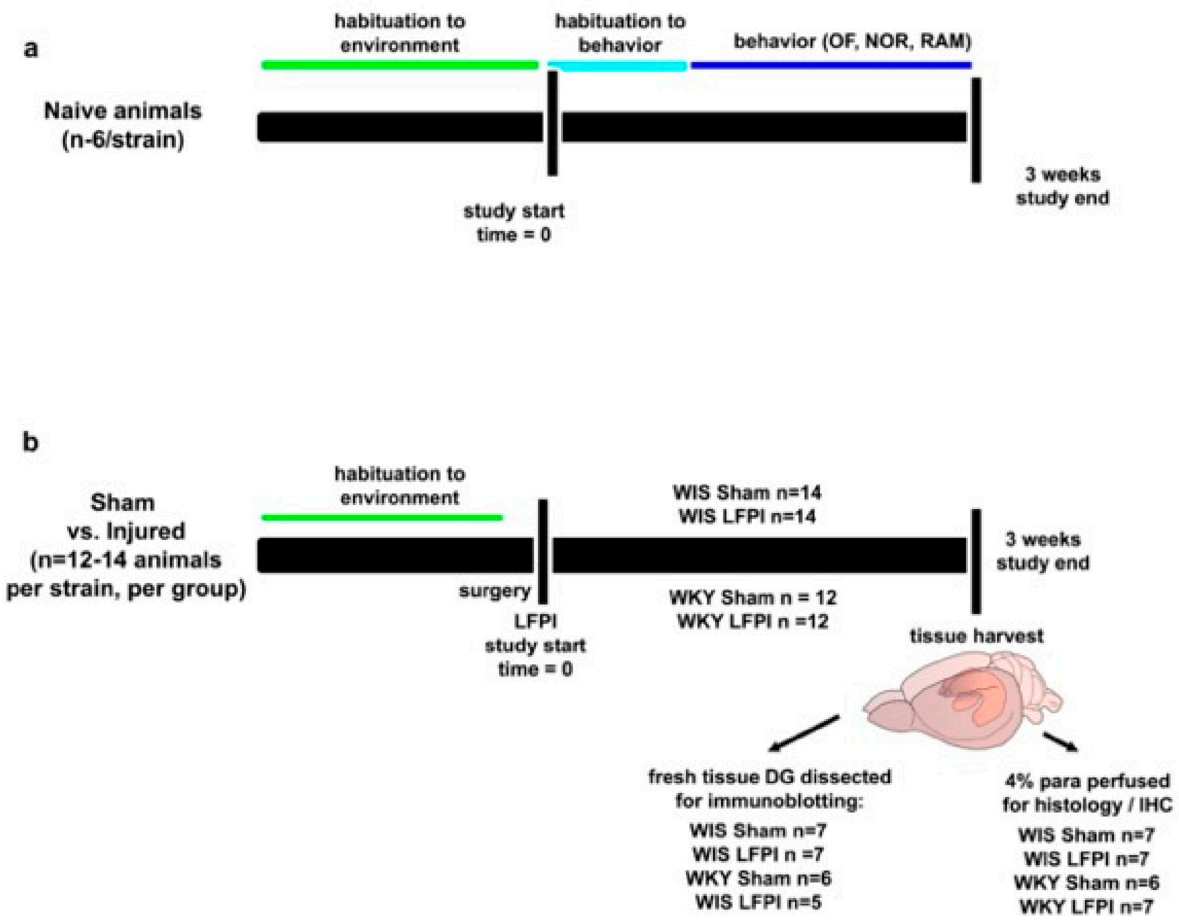

Supplementary Methods

Three animals had evidence of inappropriate injury noted at the time of tissue acquisition therefore data from these animals was not included in data analysis. One WKY sham animal had a retained anchoring screw and evidence of obvious brain bruising at time of sacrifice, therefore data from this animal was excluded. One WKY LFPI animal had evidence of contralateral injury at the site of anchoring screw placement and was excluded. One WIS sham had evidence of contralateral injury at the screw location and additional injury at the craniectomy site, suggesting penetration of the dura and/or injury at the time of surgery, this animal was excluded from further analysis.
